# A Process Systems Engineering Approach to Model and Optimize Cr^6+^-Free and Pd-Free Plating on Plastics Technologies

**DOI:** 10.3390/polym18080919

**Published:** 2026-04-09

**Authors:** Konstantinos A. Pyrgakis, Eleni Poupaki, Michalis Kartsinis, Melina Psycha, Alexios Grigoropoulos, Dimitrios Zoikis-Karathanasis, Alexandros Zoikis-Karathanasis

**Affiliations:** 1 EXELISIS, Leof. Dekelias 215 & Skra 2, 14342 Athens, Greece; melina.p@exelisis.gr (M.P.); alexis.g@exelisis.gr (A.G.); dimitris.k@exelisis.gr (D.Z.-K.); 2 Department of Mechanical Engineering, University of West Attica, 12241 Athens, Greece; 3 Creative Nano, 43 Tatoiou Str., Metamorfosi, 14451 Athens, Greecem.kartsinis@creativenano.gr (M.K.); 4 Department of Applied Physics and Science Education, Eindhoven University of Technology, 5600 MB Eindhoven, The Netherlands

**Keywords:** Plating on Plastics, etching, ABS, SSbD, materials, coatings, process design, optimization, Decision Support Tool, property models

## Abstract

Plating on Plastics (PoP) requires specific surface pre-treatment steps to enable metallization. The conventional PoP industry utilizes hexavalent chromium (toxic, carcinogenic) and palladium (critical raw material) for surface etching and activation, respectively, raising significant health, environmental, and economic concerns. This work is based on a new Cr^6+^-free and Pd-free PoP technology that uses piranha (H_2_O_2_-H_2_SO_4_) solutions for surface etching, nickel salts for activation, and NaBH_4_ for reduction, ultimately forming metallic nucleation sites for downstream electroless plating and electroplating. A comprehensive modeling approach was developed to simulate and predict unit operation performance (reaction kinetics and yields) and material properties (contact angle and adhesion) across processing stages of the new technology. State-of-the-art and data-driven modeling revealed the combinatorial relationships among process performance, the achieved properties and the different settings of process operating conditions. The results also highlighted capabilities for tuning all processes over a range of conditions, reaching desired product specifications (adhesion and thickness). The models were constructed as a Decision Support Tool (DST) serving economic, environmental, safety and Safe and Sustainable by Design (SSbD) objectives. The DST can be used through a user-friendly interface that enables the insertion of user-defined inputs and monitoring of optimization results.

## 1. Introduction

The Plating on Plastics (PoP) industry is continuously growing due to the exceptional properties of plastics (low cost and density, and easy machinability), which can be protected by a metallic coating to improve plastics’ wear and corrosion resistance and for aesthetic purposes. Since electrodeposition of metals on non-conductive plastics (e.g., ABS, PET, PLA) is not feasible, chemical pre-treatment is necessary prior to electroplating and is critical to ensure adhesion of a metallic layer on the plastic surface [1]. The conventional industrial practice first includes etching of the plastic surface with Cr^6+^-containing solutions to oxidize C=C double polymeric bonds, to form hydrophilic hydroxyl (-OH) and carboxyl (-COOH) groups and to increase surface area [1]. Secondly, Pd/Sn colloids are used for surface functionalization, catalyzing the chemical adsorption of Pd^2+^ by hydrophilic groups and Pd^2+^ reduction (Pd^2+→^Pd^0^) in the same bath. Finally, electroless plating is catalyzed by the surface Pd^0^ nucleation sites forming a first thin metallic layer, which next enables electroplating of the final metallic layer of the plastic [2]. However, there are significant concerns about the use of toxic and carcinogenic Cr^6+^ [3,4] and of Pd, a critical raw material [5], requiring alternative agents and processes capable of achieving the same, or even better, coating performance and adhesion. This work proposes a process systems engineering modeling approach to analyze and return optimal adhesion and coating performance based on a new environmentally friendly Cr^6+^-free and Pd-free PoP technology.

Unlike the advancements in the literature—these either focus on the replacement of Cr^6+^ with MnO_2_, KMnO_4_ or H_2_O_2_ oxidizing agents [6,7,8,9,10] or the replacement of Pd with cheaper Ag [2,11]—the core technology of this work addresses major safety, environmental, and economic concerns by simultaneously replacing Cr^6+^ with piranha solutions (H_2_O_2_-H_2_SO_4_) and Pd with nickel salts offering a double benefit for the PoP industry. However, the introduction of the increased complexity of multiple systems and chemistries in the PoP production lines raises critical questions about the design, scaling up and tuning of unit operations to meet the operational needs of plating facilities. In other words, new adaptive models and plug-and-play tools are required to capture the underlying physicochemical phenomena, to quantify their impacts on coating properties and to return technology design propositions quickly and robustly, ensuring maximum performance.

The formation of surface hydrophilic groups via piranha etching is crucial to next catalyze surface functionalization with nickel salts and eventually ensure high adhesion levels of the metallic coating on the plastic substrate. Moreover, the contact angle of etched plastics constitutes a key measure of the surface hydrophilicity (namely, the surface concentration of hydrophilic groups), indicating the maximum feasible number of potential anchoring points for the formation of metallic nuclei. In this scope, the existing literature does not offer systematic and evidence-based correlations of surface chemistry (hydrophilic groups), with the contact angle and adhesion properties as a function of implemented process operating conditions.

The existing literature either uses data-intensive models (e.g., thermodynamic-based) that require high precision and demanding analytical data that are hard to find and implement at experimental and industrial levels, or uses detailed computational simulation models (e.g., Computational Fluid Dynamics-CFD) that are not accessible to end-users and are unable to serve fast screening of alternative design settings. Data-demanding models use surface tensions across gas–liquid–solid phases for the prediction of contact angle and droplet stability [12], while other models use CFD [13,14] or surface-energy models discretized with finite differences [15]. Statistical analyses of adhesion in metal-based PLA implants were conducted to optimize coating parameters [16]; however, the technology is not directly relevant to the chemistries used by the PoP industry. Combinatorial insights between the contact angle and adhesion of nickel coating on ABS substrates can be inferred through the experimental data of Chen et al. [7], while the results of Zheng et al. [17] and of Wang and Zhang [18] revealed valuable indications about the relationship between contact angle and surface concentration of hydroxyls groups through surface tension and molecular dynamics simulations. However, the input data required by literature models remains out of the scope of unit operations and cannot be directly integrated into a process design framework (e.g., the optimization of piranha concentration, the etching time or the adhesion prediction).

This work solves practical challenges in modeling unconventional data relationships (like contact angle with adhesion, or hydrophilicity with formed nucleation sites) to systematically address the combinatorial insights between input operating conditions and output design variables and properties. In this scope, regression-based, data-driven and semi-mechanistic modeling concepts were exploited to explain etching, activation and reduction phenomena and to build unit operation and property prediction models. Conversely, the property prediction models can drive tuning of unit operations (e.g., baths concentrations, operating times, and applied currents) to ensure desired properties and optimal performance in terms of materials and energy used. To support decision-making and optimal unit operations, this work adopts a three-level performance framework that integrates environmental, safety, and economic criteria toward a holistic assessment. The performance criteria are apparently related to the operating conditions of the PoP process and are considered as objectives of an optimization model that is subject to the developed unit operations and property estimation equations. Finally, the optimization model was constructed as a user-friendly Decision Support Tool (DST) that receives user-defined specification targets and production requirements and returns optimal operating conditions and design settings to be used for everyday planning of operations, thus facilitating the smooth introduction of the new technology in the PoP industry.

## 2. Materials and Methods

### 2.1. Plastic Substrates Used in the Study

**Figure 1:**
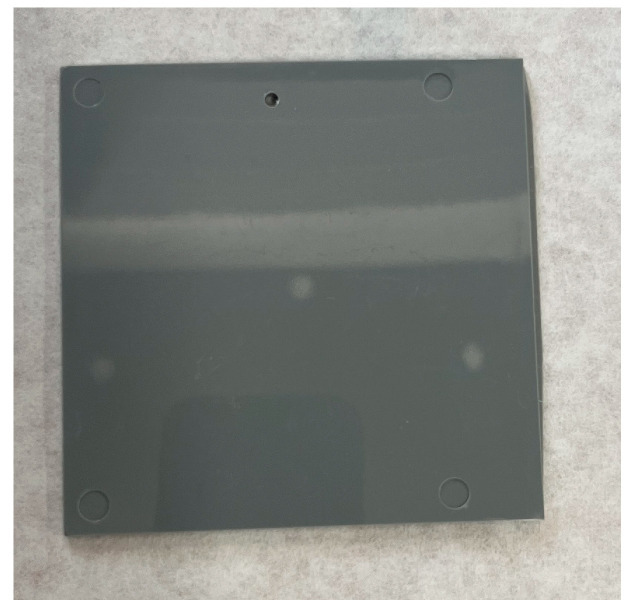
ABS samples used in this study.

The polymer substrates used in this study were commercial flat plates manufactured by injection molding from plating-grade acrylonitrile–butadiene–styrene (ABS) material. Specifically, LG ABS MP220 supplied by LG Chem Ltd. (Seoul, Republic of Korea) was used due to its suitability for metallization and electroplating applications. Rectangular samples (dimensions: 50 × 30 × 2 mm) were used in all experiments, as shown in Figure 1.

### 2.2. Overview of Novel PoP Technology

**Figure 2:**
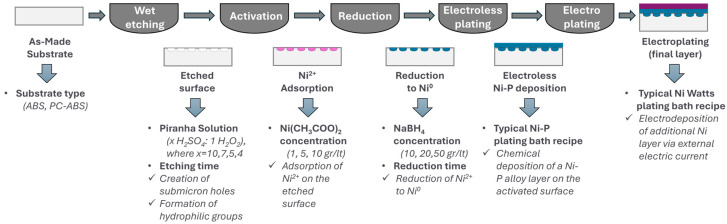
Processing steps and key design variables of the new Cr^6+^-free and Pd-free technology. Surface structure: Initial ABS substrate (grey rectangular); Etched surface (white holes); Ni^2+^ activated sites (pink holes); Reduced Ni^0^ sites (deep teal holes); Electroless nickel plated surface (deep teal layer); Electroplated surface with Nickel or other metal (purple layer).

The novel technology is illustrated in Figure 2, where a Cr^6+^-free etching process was developed using piranha solutions (H_2_O_2_-H_2_SO_4_) for the formation of interconnected cavities and hydrophilic groups on the surface. The concentration of piranha solution (H_2_O_2_-H_2_SO_4_-H_2_O) and the etching time constitute key operating conditions to ensure high etching performance; namely, determine the quality of cavities and the surface concentration of hydrophilic groups. Next, a Pd-free activation process uses nickel acetate (Ni(CH_3_COO)_2_) to catalyze the chemical adsorption of Ni^2+^ (instead of Pd^2+^), functionalizing the plastic substrate. An additional advantage of this step is that the chemical nature of the nucleation sites (here is nickel) does not restrict the type of the final metallic layer, which could be Ni, Cu, Au, or other metals. The nickel acetate concentration in the activation bath is crucial for effective chemisorption of nickel ions by the hydrophilic groups. Since surface functionalization takes place in one step, an additional processing step is required for the reduction of nickel ions (Ni^2+^) into their metallic state (Ni^0^). The concentration of the reducing agents (NaBH_4_) and the immersion time define the extent of nickel reduction toward the formation of metallic nucleation sites. An electroless Ni-P plating step is next implemented for the development of the first conductive and homogeneous thin metallic layer (~5 μm) on the plastic substrate. No degrees of freedom are identified for this step in terms of optimizing coatings’ performance, since typical electroless recipes are considered. Finally, one or more electroplating steps are applied for the development of intermediate and final coating layers [19]. In this study, one electroplating step was considered for nickel plating, where the applied current and time constitute key operating parameters to achieve the desired coating thickness.

### 2.3. Etching Processing Stage

**Figure 3:**
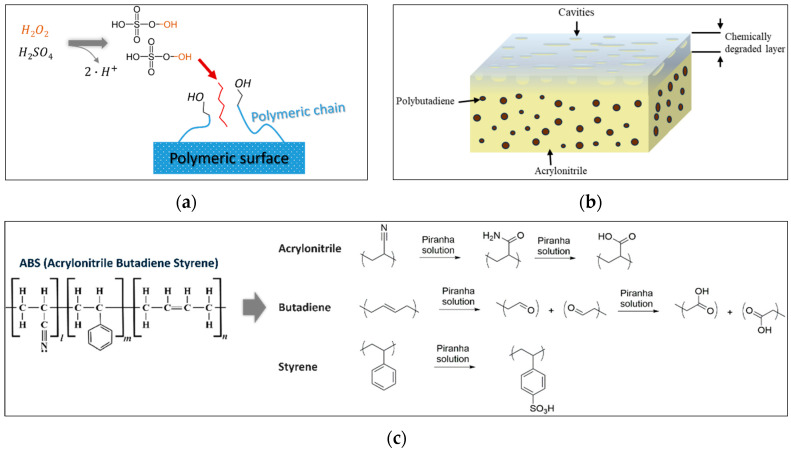
(**a**) Piranha activity on polymeric surface; (**b**) illustration of polymer surface chemical degradation; (**c**) potential reaction mechanisms of piranha with ABS monomers.

A degreasing procedure [20,21] precedes the etching process to remove dust and undesired particles from the plastic surface that may negatively affect etching performance and contamination of the etching bath. The plastic matrix was immersed in an ultrasonication bath filled with a degreasing aqueous solution composed of commercial cleaning soap (15% anionic surfactants) for 5 min. The substrate was then rinsed with reverse osmosis water for the removal of the remaining soap from the surface. Next, the etching process took place to modify the chemical nature and morphology of the polymeric surface. The process took place in a 200 mL acidic bath containing a piranha solution (H_2_O_2_-H_2_SO_4_) as strong oxidizer catalyzing the cleavage of double polymer bonds and formulating the desired cavities similarly to how Cr^6+^ functions (Figure 3a). Etching was performed at room temperature (25 °C), within piranha solutions ranging from 1:4–1:10 (H_2_O_2_-H_2_SO_4_) and for operating times within 10–180 s. This experimental design covered a wide range of operating conditions, catching the whole spectrum of reaction phenomena from intensive etching (at 1:4) to moderate and smooth etching (at 1:10). Rinsing with reverse osmosis water followed each stage, including etching, activation, reduction, electroless plating and electroplating.

Cleaving of double bonds results in the creation of -COOH and -OH hydrophilic groups at the free open edges of the polymer chain. These groups constitute the precursor anchoring points between the polymeric surface and the metallic layer. The cleavage of butadiene bonds also spread inside the polymer body as spheres, resulting in the formation of micro- and nano-sized cavities (Figure 3b), improving the surface concentration of hydrophilic groups and enhancing the mechanical adhesion of the metallic layer. The set of key etching reactions of Figure 3c was considered for piranha etching of ABS. Intensive and uncontrolled etching could result in polymer degradation and poor adhesion of the coating. For this reason, the optimization of the etchant concentration and the etching time plays a critical role in reaching the desired surface properties, and thus, this work prepares a model to address such trade-offs and identify the best etching strategies.

### 2.4. Activation Processing Stage

**Figure 4:**
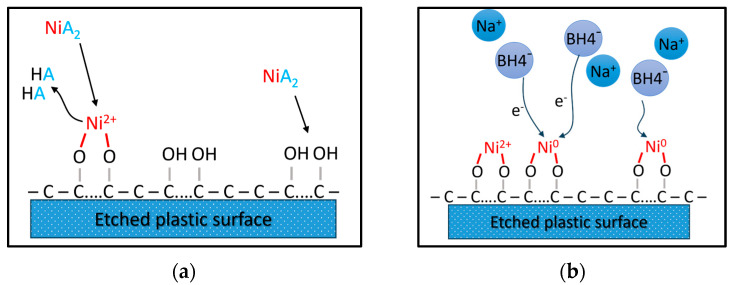
(**a**) Activation of etched surface with nickel acetate; (**b**) reduction of activated surface with NaBH_4_.

The activation step builds the surface metal nucleation sites through immersion of the etched substrate into salt solutions using a 1st-row transition metal (e.g., Pd, Ni, or Cu). Several groups in the literature have proposed using Ni(II) salts, like NiSO_4_ [22] or Ni(CH_3_COO)_2_ [20,23]. In this work, nickel acetate (Ni(CH_3_COO)_2_) was used to catalyze the chemisorption of Ni^2+^ by hydrophilic groups of the etched surface toward the formation of nickel-based nucleation sites (Figure 4a), while the chemical identity of nucleation sites does not affect the selection of the final metallic coating. The activation stage was performed at 45 °C for three different nickel acetate concentrations (1, 5 and 10 g/L) and for 30 min, ensuring enough time for the system to reach adsorption equilibrium. A uniform distribution of the metal cations is desired to facilitate (at a next stage) the homogeneous growth of the metal coating.

### 2.5. Reduction Processing Stage

In the case of the Pd/Sn colloid, both chemisorption and reduction of Pd are conducted in a one-step process. By contrast, in this work, where nickel cations were used for surface activation, a separate reduction step is required during which the sample is immersed in an aqueous solution of a strong reducing agent. In this stage, the adsorbed nickel cations are reduced (Figure 4b) to their metallic state, Ni^0^, forming the required nucleation sites where the metal coating will be chemically deposited in the following electroless step. The most common reducing agents are sodium and potassium borohydride (NaBH_4_, KBH_4_) [8,22,23]. In this work, NaBH_4_ was selected due to its well-established reducing power (E0 = −1.24 V vs. standard hydrogen electrode), its capability to reduce nickel cations to elemental nickel, and its relatively low price compared to the other reducing agents. The reduction stage was tested at room temperature (25 °C) and at three different concentrations (10, 20, 50 g/L) and reaction times (1, 5, 10 min).

### 2.6. Electroless Plating

**Figure 5:**
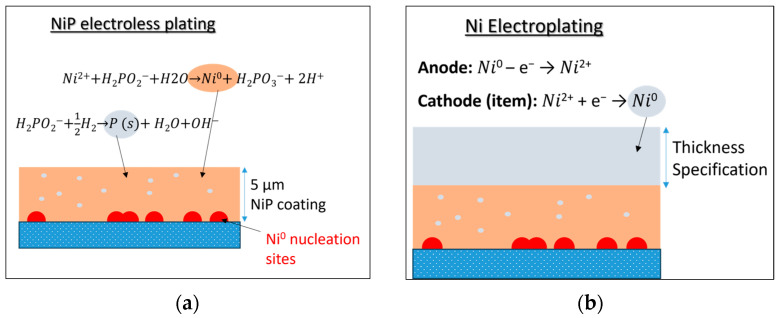
(**a**) Electroless plating of reduced substrate; (**b**) electroplating of the electroless plated substrate.

The next stage involves the chemical deposition of a Ni-P alloy layer (Figure 5a) on the reduced surface by immersing the sample into a nickel–phosphorus electroless bath, developing the first metallic layer in the absence of electric current. The typical recipe of Table 1 was implemented by including the metal salt (NiSO_4_·6H_2_O), a reducing agent (NaH_2_PO_2_) acting as an electron source for deposition, a complexing agent for the metal (Na_3_C_6_H_5_O_7_·2H_2_O), and a compound (NH_4_OH) for pH adjustment. Electroless plating forms a uniform, lustrous and conductive thin metallic layer of 5 μm on the substrate’s surface within 30 min of immersion. Besides nickel, other metals such as copper, gold or platinum can be used for electroless plating by altering the reducing agent (e.g., organoboron, hydrazine, formaldehyde); thus, new alloys or pure metal coatings can be achieved.

### 2.7. Electroplating

The conductivity of the Ni-P layer allows the deposition of extra metallic layers (e.g., Au, Cu and Ni) via electroplating to achieve the final desired surface properties (appearance, chemical resistance, mechanical strength, electromagnetic shielding, etc.). The standard nickel Watts electroplating bath of Table 2 was used to produce a pure nickel coating on top of the electroless Ni-P layer. The applied current density ranged within 1.3–1.7 A/dm^2^, and the plating time ranged within 0.5–2 h. A typical electroplating setup was utilized through an electrolyte solution to reduce dissolved metallic cations to their corresponding metallic atoms, which are then deposited on the substrate’s surface. The Ni-P plated substrate is implemented as the cathode of the electrolytic cell, while a solid block of Ni metal is used as the anode [24]. The aqueous solution contains the metallic cations and chemical compounds that improve the quality of the final coating and efficiency of the electroplating process. These compounds include weak acids or bases as buffering agents, organic additives (surfactants, brighteners, stress relievers and metal complexing agents) or even inorganic nanoparticles that improve mechanical properties. The type and the number of the electroplating steps may vary based on the specific end-user needs.

### 2.8. Safety Considerations

Special safety considerations are required for the preparation and handling of piranha solutions, which are highly oxidative and strongly exothermic upon mixing. In the present work, H_2_O_2_ was slowly added to H_2_SO_4_ immediately prior to use, while all treatments were conducted in open glass containers under appropriate ventilation and temperature monitoring to prevent uncontrolled reactions. Extra caution was taken to avoid contact of hot piranha solutions with organic materials, which may cause rapid heat and gas evolution, leading to hazardous environments. Personal protective equipment, including a face shield, chemical-resistant gloves and protective clothing, was used during all processing steps. After use, spent etching baths were allowed to cool and were subsequently neutralized through controlled dilution and alkaline treatment to reach near-neutral pH prior to disposal in accordance with applicable environmental regulations. During the reduction stage, NaBH_4_ solutions were handled under controlled conditions and adequate ventilation due to their strong reducing character and potential hydrogen evolution. After use, residual NaBH_4_ solutions were neutralized by controlled dilution followed by gradual acidification to ensure safe hydrolytic decomposition prior to disposal in accordance with applicable environmental and safety regulations.

## 3. Results

### 3.1. Property and Unit Operation Models

#### 3.1.1. Contact Angle Property Prediction

**Figure 6:**
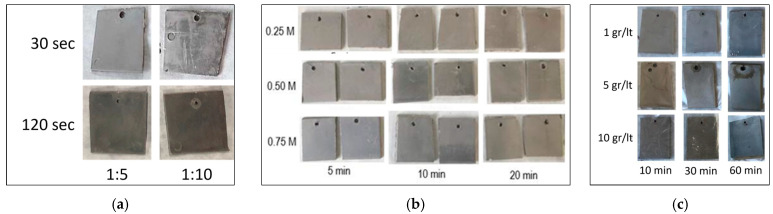
Samples’ images after (**a**) etching, (**b**) activation and (**c**) reduction of ABS.

The contact angle was considered as an equivalent measure of the surface concentration of hydrophilic groups reflecting the activity of piranha solution in the cleavage of polymeric bonds and the extent of etching reactions. The experimentally measured contact angles of each etched ABS substrate for different piranha solutions and etching times are presented in Table 3. Moreover, the initial contact angle of the untreated ABS substrate was measured at 86.67°. To ensure reproducibility and reliability of the experimental data, each experimental condition was repeated at least 3 times, and the reported values correspond to the mean measurements. This practice was followed for all experimental tests performed for etching, activation, reduction, and plating steps. The experimental variability was evaluated through the calculation of standard deviation for key parameters, including contact angle, coating adhesion and deposited mass. Instrumental uncertainties were considered according to the specifications of the measurement devices used (e.g., contact angle goniometer and adhesion testing system). Outliers were identified through consistency checks across repeated measurements and were excluded only when clear experimental anomalies were observed, such as measurement instability or sample defects. This approach was adopted to improve the robustness of the developed regression and kinetic models. The etching performance at different piranha concentrations and etching times can also be visually assessed using the sample images in Figure 6 after each step. In contrast, visual identification of differences among plated samples is limited due to the similarity of samples with similar outer metal finishes.

Given the dataset of Table 3, a regression-based model has been developed to predict the contact angle of etched plastics as a function of the piranha concentration and the etching time. The model indirectly embeds the reaction mechanisms of piranha on ABS substrates, in a similar sense that the etching kinetic model does; only that this model predicts contact angle instead of the actual hydrophilic groups concentrations.

Two main mechanisms have been considered regarding the reaction of H_2_O_2_ with H_2_SO_4_, and their etching activity, where both mechanisms result in the formation of oxygen (O∙) and hydroxyl (∙HO) radicals featuring the oxidative nature that alters the nature of the plastic surface. One mechanism (Equation (1)) is based on the formation of Caro’s acid (H_2_SO_5_), which further decomposes into ∙HO radicals. The other mechanism (Equation (2)) produces hydrated hydrogen ions and atomic oxygen (O∙), whose activity is crucial in cleaving polymeric C=C bonds toward the formation of hydroxyl and carboxylic groups on the polymer’s edges [25,26,27].

H_2_SO_4_ + H_2_O_2_ ⇌ H_2_SO_5_ + H_2_O → H_2_O + HSO_3_O∙ + ∙HO (1)

H_2_SO_4_ + H_2_O_2_ → H_3_O^+^ + HSO_4_^−^ + O∙ (2)

Both mechanisms are driven by a 1:1 stoichiometry for H_2_O_2_ and H_2_SO_4_. In all experiments (Table 3), an excess of H_2_SO_4_ was utilized (>7.4 moles H_2_SO_4_ per mole of H_2_O_2_), ensuring that the containing H_2_O_2_ molecules are adequately utilized toward the formation of the required O∙ and ∙HO radicals driving etching reactions. As a result, a H_2_O_2_ concentration was selected as the first key regression variable of the etching model, while etching time was the second key reaction variable.

For the modeling of contact angle, this work was inspired by the DIPRR EQ101 formulation—that is $f\left(x\right)=exp\left(A+\frac{B}{x}+C\cdot ln\left(x\right)+D\cdot x^{E}\right)$—due to its efficiency in adopting complex behaviors by incorporating different non-linear and adaptive terms. Based on DIPRR EQ101, contact angle (CA) was first expressed as a function of time (t) as:

(3) $$CA=exp\left(A+\frac{B}{\left(t+1\right)}+C\cdot ln\left(t+1\right)+D\cdot {\left(t+1\right)}^{E}\right)$$

where time is embedded in the form of (t+1) to ensure validity of the equation at t=0 s, while A, B, C, D,E>0 are variables that incorporate the effects of [H_2_O_2_] and the nature of the material. Key considerations for Equation (3) include (i) negative first derivative due to decreasing CA with time, and (ii) CA is equal to the contact angle of the untreated polymer (${CA}^{0}$) at t=0 s. As a result:

$$CA^{\prime }=CA\cdot \left[\frac{-B}{{\left(t+1\right)}^{2}}+\frac{C}{t+1}+D\cdot E\cdot {\left(t+1\right)}^{E-1}\right]<0\overset{\;⠀}{\Rightarrow }\frac{-B}{{\left(t+1\right)}^{2}}+\frac{C}{t+1}+D\cdot E\cdot {\left(t+1\right)}^{E-1}<0\overset{\;⠀}{\Rightarrow }$$

(4) $$B>\left(t+1\right)\cdot \left[C+D\cdot E\cdot {\left(t+1\right)}^{E}\right]$$

If C, D,E>0, then Equation (4) implies that B strictly increases with time $\left(\underset{t\to \infty }{\lim}B=\infty \right)$, which is not valid, since B is independent of time and is fixed by fixing [H_2_O_2_] and the type of material. As a result, the right part of Equation (4) is redefined resulting in zero—either due to C=0 and D=0 or C=0 and E=0—and thus, Equation (4) reduces in the form of:

(5) $$CA=exp\left(A+\frac{B}{\left(t+1\right)}\right)\;\;\;or\;\;\;CA=exp\left(A+\frac{B}{\left(t+1\right)}+D\right)$$

At t=0 s, $CA={CA}^{0}$, and Equation (5) returns:

(6) $${CA}^{0}=exp\left(A+B\right)\;\;\;or\;\;\;{CA}^{0}=exp\left(A+B+D\right)\overset{\;⠀}{\Rightarrow }A=ln\left({CA}^{0}\right)-B\;\;\;or\;\;\;A=ln\left({CA}^{0}\right)-B-D$$

By replacing A in Equation (6) in Equation (5), both forms of Equation (5) result in the common final form of:

(7) $$CA={CA}^{0}\cdot exp\left(\frac{-B\cdot t}{t+1}\right)$$

Next, the DIPPR EQ101 was also used (for the same reasons) for the expression of B as a function of [H_2_O_2_] and the type of material as:

(8) $$B=exp\left(A^{\prime }+\frac{B^{\prime }}{\left[H_{2}O_{2}\right]}+C^{\prime }\cdot ln\left(\left[H_{2}O_{2}\right]\right)+D^{\prime }\cdot {\left(\left[H_{2}O_{2}\right]\right)}^{E^{\prime }}\right)$$

where the parameters $A^{\prime },B^{\prime },C^{\prime },D^{\prime },E^{\prime }$ are material-dependent and [H_2_O_2_] is defined by the concentration of the piranha solution, without excluding the option of water dilution of piranha. Equation (8) is subjected to an additional condition according to which there is an optimal piranha solution ($\bar{C}$) where contact angle reaches a minimum value at theoretically infinite etching time. This assumption is valid, since below $\bar{C}$ etching becomes less effective, while above $\bar{C}$ etching starts being aggressive, resulting in uncontrolled etching. Based on experimental data of Table 3, the lowest contact angle is observed at 1:5 piranha solution, which is $\bar{C}=1.62$ mol/L. The first derivative of CA by [H_2_O_2_] should be equal to zero at $\left[H_{2}O_{2}\right]=\bar{C}$.

(9) $$\frac{dCA}{d\left[H_{2}O_{2}\right]}|\begin{array}{c} \;⠀\\ \left[H_{2}O_{2}\right]=\bar{C} \end{array}=0$$

Based on Equation (7) and considering the effects of [H_2_O_2_] through Equation (8), Equation (9) is transformed into:

(10) $${CA}^{0}\cdot exp\left(\frac{-Bt}{\left(t+1\right)}\right)\cdot \left(-\frac{t}{\left(t+1\right)}\right)\cdot B\cdot \left(\frac{-B^{\prime }}{{\bar{C}}^{2}}+\frac{C^{\prime }}{\bar{C}}+D^{\prime }E^{\prime }\cdot {\bar{C}}^{E^{\prime }-1}\right)=0$$

Since all terms of Equation (10) are non-zero except for the last one, these terms must be equal to zero as follows:

(11) $$\frac{-B^{\prime }}{{\bar{C}}^{2}}+\frac{C^{\prime }}{\bar{C}}+D^{\prime }E^{\prime }\cdot {\bar{C}}^{E^{\prime }-1}=0\overset{\;⠀}{\Rightarrow }B^{\prime }=C^{\prime }\cdot \bar{C}+D^{\prime }E^{\prime }\cdot {\bar{C}}^{E^{\prime }+1}$$

Equation (11) explains that at least one parameter of Equation (8) can be eliminated, and thus, Equation (8) is finally transformed into:

(12) $$B=exp\left(A^{\prime }+\frac{C^{\prime }\cdot \bar{C}+D^{\prime }E^{\prime }\cdot {\bar{C}}^{E^{\prime }+1}}{\left[H_{2}O_{2}\right]}+C^{\prime }\cdot ln\left(\left[H_{2}O_{2}\right]\right)+D^{\prime }\cdot {\left(\left[H_{2}O_{2}\right]\right)}^{E\prime }\right)$$

The overall CA model comprises Equations (7) and (12), where the parameters ${CA}^{0}$, $\bar{C}$, $A^{\prime }$, $C^{\prime }$,$\;D^{\prime }$ and $E^{\prime }$ are material-dependent—with ${CA}^{0}$ and $\bar{C}$ being experimentally defined—while t and [H_2_O_2_] constitute the independent variables of the etching process. The CA model (Equations (7) and (12)) was fitted with $R^{2}=0.9647$, yielding parameter estimation as follows: $C_{A}^{0}=86.67$, $\overset{ˉ}{C}=1.62$, $A^{\prime }=86.30$, $C^{\prime }=-38.94$, $D^{\prime }=552.29$, and $E^{\prime }=-1.09$.

#### 3.1.2. Surface Concentration

**Figure 7:**
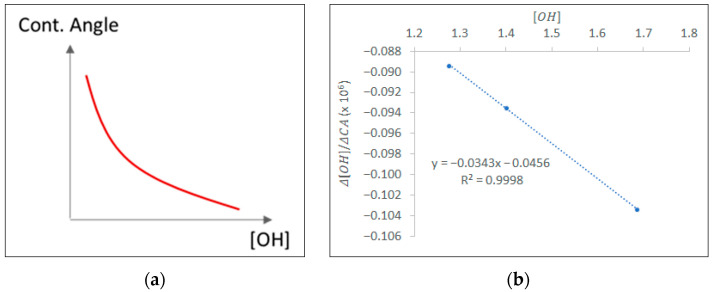
(**a**) Correlation between surface concentration of hydrophilic groups [OH] with contact angle (CA); (**b**) fitted data relating [OH] with CA.

As mentioned in the literature [22,28,29], the contact angle is considered inversely proportional to the surface concentration of hydrophilic groups, as illustrated in Figure 7a. To model this effect—namely, contact angle approaching a minimum feasible value (${CA}^{min}$) at maximum hydrophilic group concentration [OH]—the following model was initially devised:

(13) $$\frac{dCA}{d[OH]}=k1\cdot \left(CA-{CA}^{min}\right)$$

When Equation (13) is integrated at boundary conditions ($CA={CA}^{0}$ at [OH]=0), then Equation (14) is returned, which is next used (Equation (15)) to relate [OH] between two different states (1 and 2) as a function of the respective contact angles measured in each state. Equation (15) is powerful since it reveals significant insights regarding [OH].

(14) $$ln\left(\frac{CA-{CA}^{min}}{{CA}^{0}-{CA}^{min}}\right)=k1\cdot \left[OH\right]$$

(15) $$\frac{ln\left(\frac{{CA}^{2}-{CA}^{min}}{{CA}^{0}-{CA}^{min}}\right)}{ln\left(\frac{{CA}^{1}-{CA}^{min}}{{CA}^{0}-{CA}^{min}}\right)}=\frac{{\left[OH\right]}^{2}}{{\left[OH\right]}^{1}}$$

Equation (15) was used to model contact angle before (state 1) and after (state 2) activation, while ${CA}^{min}=\;$27.82° and ${CA}^{0}$ = 86.67°. Thus, ${CA}^{1}$ was used for the contact angle of the etched substrate and ${CA}^{2}$ for the activated substrate. It is assumed that after enough activation times (>120 min), an equilibrium is reached by the system. This assumption is also experimentally observed, since contact angle fluctuations during activation are suppressed at high processing times, and the contact angle converged. Given the total activation reaction, ${NiA}_{2}+2\;COH\to CONiOC+2\;HA$, where ${NiA}_{2}$ is nickel acetate, COH are the free hydroxy/carboxyl groups that adsorb Ni^2+^, CONiOC reflects the formulation of adsorbed Ni^2+^ and HA is the hydrogenated acetate group, the equilibrium can be developed as:

(16) $$K^{eq}=\frac{\prod {\left[products\right]}^{\nu }}{\prod {\left[reactants\right]}^{m}}=\frac{a\cdot a^{2}}{\left(\left[{NiA}_{2}\right]-a\right)\cdot {\left({\left[OH\right]}^{2}\right)}^{2}}$$

where $\left[{NiA}_{2}\right]$ is the initial concentration of the activation bath [mol/L]; a is the amount reacted ${NiA}_{2}$ (mol/L); ${\left[OH\right]}^{1}$ and ${\left[OH\right]}^{2}$ are the initial and final surface concentrations of hydroxyl groups before and after activation (mol/cm^2^); and $K^{eq}$ is estimated by ${{\Delta G}^{0}}_{react}=-RT\cdot ln\left(K^{eq}\right),\;\;where\;{{\Delta G}^{0}}_{react}$ was calculated based on the ${\Delta G}_{comp}^{0}$ of reaction components provided by property libraries and the use of group contribution methods.

Provided that ${\left[OH\right]}^{2}={\left[OH\right]}^{1}-2\cdot a$, Equations (15) and (16) are solved toward the estimation of ${\left[OH\right]}^{1}$ and a. Furthermore, by solving Equations (15) and (16) for different activation conditions (e.g., $\left[{NiA}_{2}\right]$ = 1, 5 or 10 g/L), different hydroxyl concentrations before activation ${\left[OH\right]}^{1}$ can be estimated. This is inconsistent given that ${\left[OH\right]}^{1}$ is a characteristic feature of the etched substrate. However, the different ${\left[OH\right]}^{1}$ values appear a second-order relationship with $\frac{{\left[OH\right]}^{2}}{{\left[OH\right]}^{1}}$ in the form of $\frac{{\left[OH\right]}^{2}}{{\left[OH\right]}^{1}}=a1\cdot ln\left({\left[OH\right]}^{1}\right)+a2$, where a1=−0.081 and a2=−0.040 are fitting parameters. This expression is used to correct and align the different ${\left[OH\right]}^{1}$ predictions. In the absence of activation, $\frac{{\left[OH\right]}^{2}}{{\left[OH\right]}^{1}}=1$, and, thus ${\left[OH\right]}^{1}=e^{\frac{1-a2}{a1}}$, which returns a single ${\left[OH\right]}^{1}$ value for the etched plastic. Based on the corrected ${\left[OH\right]}^{1}$, ${\left[OH\right]}^{2}$ can be estimated through Equation (15), and the results are summarized in Table 4.

Given the results of Table 4, the initially considered Equation (13) can be revised, taking advantage of the linear relationship that was observed in Table 4 for $\frac{d\left[OH\right]}{dCA}$ as a function of $\left[OH\right].\;\frac{d[OH]}{dCA}$ can be computed as discrete differences ($\frac{\Delta [OH]}{\Delta CA}$) of the estimated [OH] and the experimentally measured CA, resulting in the satisfying linear regression (R^2^ = 0.9998) of Figure 7b and Equation (17). Integration of Equation (17) results in the final valuable correlation of [OH] with CA, which can be used to approximate the hydrophilic group’s surface concentration based on the experimentally measured contact angle.

(17) $$\frac{d[OH]}{dCA}=-0.03427\cdot \left[OH\right]-0.0000000456\overset{\int \;⠀}{\Rightarrow }ln\left(751,510\cdot \left[OH\right]+1\right)=-0.03427\cdot \left(CA-{CA}^{0}\right)$$

#### 3.1.3. Activation Process Models

**Figure 8:**
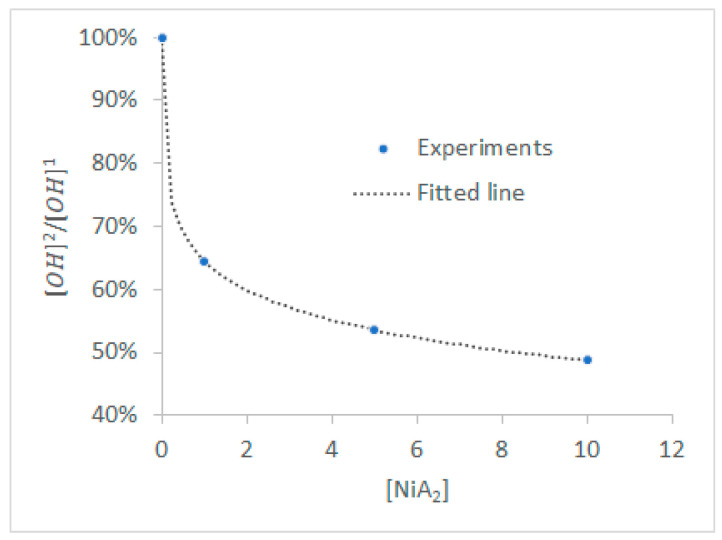
Fitted data relating [OH] before and after activation with [NiA_2_].

The purpose of the activation model is to estimate the surface concentration of adsorbed nickel, which plays a crucial role in downstream plating. Given that 2 moles of hydrophilic groups adsorb 1 mol of Ni^2+^, the concentration of nucleation sites ([CONiOC]) can be calculated as $\left[CONiOC\right]=\frac{\left({\left[OH\right]}^{1}-{\left[OH\right]}^{2}\right)}{2}$. Based on the experimental data of Table 4, a non-linear approximation was built for the estimation of $\frac{{\left[OH\right]}^{1}}{{\left[OH\right]}^{2}}$ as a function of [${NiA}_{2}$] (Figure 8). The fitted line of Figure 8 should strictly intersect the *Y*-axis at 1 to ensure that no activation occurs at [${NiA}_{2}$] = 0, and thus, the hydrophilic groups concentration remains unaffected. Equation 18 describes the fitted line of Figure 8, effectively describing activation performance as a function of activation agent concentration, with an R^2^ value approaching 1.

(18) $${\left[OH\right]}^{2}=\frac{{\left[OH\right]}^{1}}{0.5524\cdot {\left[{NiA}_{2}\right]}^{0.2791}+1}$$

#### 3.1.4. Reduction Process Kinetics

**Figure 9:**
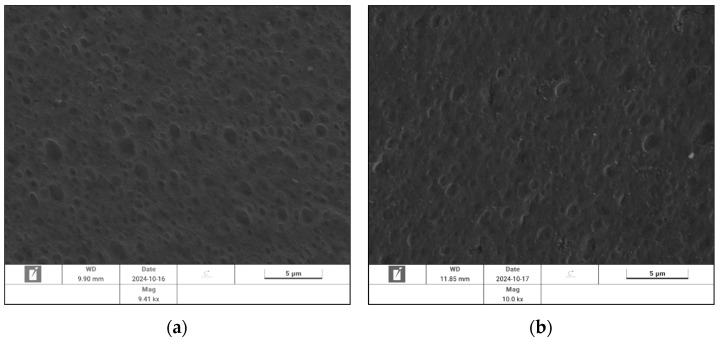
SEM/EDS analysis of (**a**) activated ABS sample (before the reduction) and (**b**) activated and reduced ABS (right after reduction).

**Figure 10:**
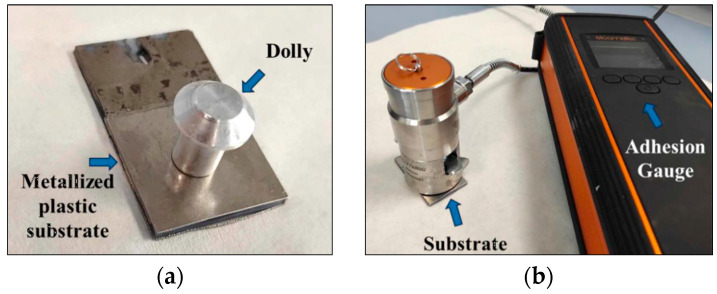
(**a**) Ni-Watts electroplated plastic substrate with glued dolly; (**b**) Mounting of the pull-off adhesion gauge to measure adhesion.

Reduction produces the final anchoring sites (Ni^0^) between the plastic and the metal layers, and the defining adhesion of the metallic layer on the plastic substrate was measured. A reduction kinetic model was developed to address the desired reduction of Ni^2+^ sites into their metallic state (Equation (19)) as well as undesired side reactions (Equation (20)), which are expected to either cover nucleation sites or limit the effectiveness of redox reactions (e.g., ${Ni}^{2+}$ and ${BH}_{4}^{-}$), thus inhibiting and limiting nickel reduction. The formation of affecting by-products can be hypothesized through SEM/EDS analyses results (Figure 9 and Table 5), where sodium from NaBH_4_ is observed on the reduced surface and through decreasing coating adhesion at higher NaBH_4_ concentrations (Table 6). There are several alternative phenomena that may explain lower adhesion due to the presence of sodium, like blocking and co-existence with nickel nucleation sites or the formation of chemical complexes and limiting redox reactions, which can inhibit the formation of Ni^0^ sites, or even limit chemical bonding of Ni^0^ anchoring sites with the Ni-P layer. Under these conditions, the balancing of reduction and side reactions is necessary to maximize adhesion benefits. Reduction of activated samples was tested at 10, 20 and 50 g/L of NaBH_4_ and at 1, 5 and 10 min of operation. Each reduced sample was plated using the same electroless and electroplating recipes and conditions, and the adhesion of the metallic layer was measured (in MPa) using the Pull-Off Adhesion Test method (Figure 10).

(19) $${Ni}^{2+}+2\;{BH}_{4}^{-}+6\;H_{2}O\to {Ni}^{0}+2\;B{\left(OH\right)}_{3}+7\;H_{2}$$

(20) $${Ni}^{2+}+{NaBH}_{4}^{\;⠀}\to H_{2}+byproducts$$

An evolutionary procedure was followed to build the terms of the reduction kinetic model of Equation (21). The model features dependencies on both reagents (Ni^2+^ and NaBH_4_) and addresses competitiveness with undesired reactions (Equation (20)) at high NaBH_4_ concentrations. It is noteworthy that $\left[NaBH_{4}\right]$ in Equation (21) refers to sodium borohydride that is consumed by the desired reaction toward nickel reduction.

(21) $$\frac{d\left(\left[{Ni}^{2+}\right]\right)}{dt}=-\frac{k2\cdot \left[{Ni}^{2+}\right]\cdot \left[NaBH_{4}\right]}{1+k3\cdot {\left[NaBH_{4}\right]}^{2}}$$

In these terms, the kinetic parameters $k_{2}$ and $k_{3}$ constitute the reaction rate constants that describe the two different aspects of the reaction mechanism. The parameter $k_{2}$ reflects the apparent reduction rate and reflects the intrinsic kinetics of the reaction, typically associated with electron transfer. In contrast, $k_{3}$ accounts for non-ideal behavior such as saturation or inhibition effects, related to adsorption phenomena, competition, intermediate species formation, or surface blocking. As a result, the model deviates from simple power-law kinetics and resembles a Langmuir type of expression. At low NaBH_4_ concentrations, the reaction follows second-order kinetics, while at higher NaBH_4_ concentrations, undesired phenomena become significant, leading to a reduction of the overall rate and a reduction of NaBH_4_ consumed for nickel reduction.

The reduction kinetic model was fitted against adhesion measurements, as an equivalent measure of the reduced nickel concentration ($\left[{Ni}^{0}\right]$), which is equal to the difference between the initial and final $\left[{Ni}^{2+}\right]$. In this scope, the surrogate model of Equation (22) was devised to relate [Ni^0^] with the adhesion (AD) property of the plated plastic substrate. The model was inspired by the Langmuir saturation model and is used to appropriately describe the convergence of adhesion to a maximum feasible value (${AD}^{max}$) as surface concentration of nucleation sites ([Ni^0^]) increases, with parameter N controlling how quickly the system can approach convergence at increasing [Ni^0^]. The highest adhesion value that was observed across all experiments (including triplicates) was ${AD}^{max}=8.85\;MPa$.

(22) $$AD=\frac{{AD}^{max}\cdot N\cdot \left[{Ni}^{0}\right]}{1+N\cdot \left[{Ni}^{0}\right]}$$

**Figure 11:**
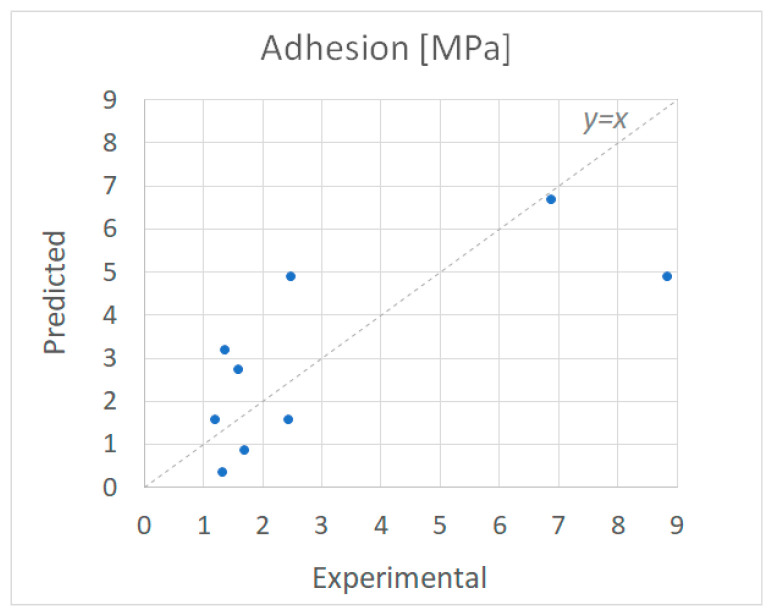
Experimentally measured and computationally predicted adhesion values.

**Figure 12:**
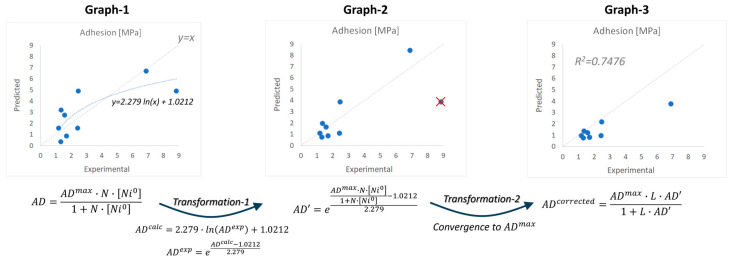
Re-mapping model showing predicted adhesion values against the experimentally measured values.

The combined model of Equations (21) and (22) was fitted against the experimental data of Table 6 to minimize the root mean square error. Figure 11 presents the fitting of the predicted adhesion values versus the experimentally measured values, while the fitting parameters were estimated at $k2=141\;\frac{lt}{sec\cdot mol}$, $k3=1,517,722\;\frac{lt}{mol}$ and $N=7,491,888\;\frac{{cm}^{2}}{mol}$. Moreover, the data processing procedure shown in Figure 12 can be further optionally implemented to re-map the calculated adhesion values of Figure 11 and improve the accuracy of the adhesion model with a more conservative perspective than that of Figure 11 to avoid overestimations of adhesion that may result in defective coatings that fall below the expected adhesion specifications.

A logarithmic trendline appears to provide the best fit for the data points in Figure 11; the trendline is presented in Graph-1 of Figure 12. An inverse function of this correlation (Transformation-1 of Figure 12) explains the correction of the calculated adhesion values to meet experimental values. Thus, an updated version of the adhesion model—this is the function ${AD}^{\prime }$ under Graph-2 of Figure 12—is developed and solved along with the reduction kinetics (Equation (21)), resulting in the new fitting of Graph-2 of Figure 12. The total absolute distance of the data points from the y = x line in Graph-2 is 8% smaller than that of Graph-1; thus, Transformation-1 improved adhesion predictions. Most distant points from the y = x line could be excluded (e.g., the point excluded in Graph-2) to further improve the total absolute distance. Finally, another transformation (Transformation-2) is required to restore the ability of the initial adhesion model to converge toward ${AD}^{max}$. For this purpose, the same modeling concept of Equation (22) is implemented for the updated adhesion model (${AD}^{\prime }$), resulting in Equation (23):

(23) $${AD}^{corrected}=\frac{{AD}^{max}\cdot L\cdot {AD}^{\prime }}{1+L\cdot {AD}^{\prime }}=\frac{{AD}^{max}\cdot L\cdot e^{\frac{AD-1.0212}{2.279}}}{1+L\cdot e^{\frac{AD-1.0212}{2.279}}}$$

where AD is calculated by Equation (22).

The final system of Equations (21)–(23) was regressed against the experimental data of Table 6, resulting in the fitting of Graph-3 in Figure 12 (R^2^ = 0.7476) and estimation of the model parameters as follows: $k2=6.67\;\frac{lt}{sec\cdot mol}$ and $k3=51,922,172\;\frac{lt}{mol}$ for Equation (21), $N=2,308,308,506\frac{{cm}^{2}}{mol}$ for Equation (22), and L=0.104 for Equation (23). The new set of the predicted adhesion values in Graph-3 of Figure 12 is significantly improved and more conservative compared with the predictions in Figure 11.

#### 3.1.5. Coating Thickness

For the estimation of the thickness of the final coating developed through electroplating, a growth rate-based model was considered for the electrodeposition of the metallic layer. The model estimates the actual plated mass (${mass}^{real}$) as a function of the theoretical mass (${mass}^{theo}$), defined based on the applied current and the plating time as follows:

(24) $${mass}^{real}={mass}^{theo}\cdot \left(1-e^{-gr\cdot t}\right)$$

where gr is the growth rate constant and t is the plating time. The theoretically maximum feasible mass that could be deposited is estimated as ${mass}^{theo}=\frac{I\cdot S\cdot t\cdot MW}{n\cdot F}$, where I is the current density (A/dm^2^), S the surface (dm^2^), Mr the molecular weight of the applied metal (58.69 g/mol for Ni), n the number of electrons per metal ion (2 for Ni) and F the Faraday constant (96,485 C/mol).

**Figure 13:**
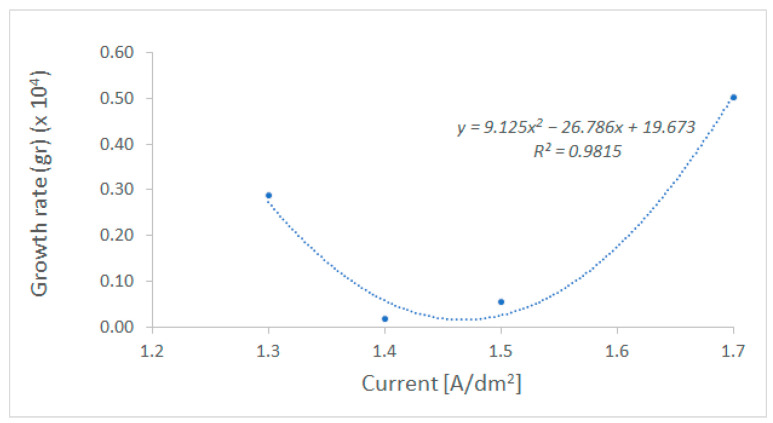
Fitted data relating electroplating growth rate with applied current densities.

Equation (24) was transformed as a function of $gr=f\left({mass}^{real}\right)$ and was fitted against the experimental data of Table 7, resulting in growth rates for different current densities and operating times. Accordingly, a second-order effect of the current density on growth rate was observed (Figure 13) and provides good fitting (R^2^ = 0.9815), resulting in the expression $gr=0.0091\cdot I^{2}+0.0268\cdot I+0.0197$. Finally, the thickness (TH) of the electroplated metallic layer can be estimated by $TH=\frac{{mass}^{real}}{\rho \cdot S}=\frac{{mass}^{theo}\cdot \left(1-e^{-\left(0.0091\cdot I^{2}+0.0268\cdot I+0.0197\right)\cdot t}\right)}{\rho \cdot S}$, where ρ is the density of the metallic layer (8.9 kg/L for nickel) and S is the surface of the substrate.

### 3.2. Optimization Framework

Based on the process and property prediction models of Section 3.1, an optimization tool was developed to simulate, scale up and optimize the design and operating conditions of all processing stages of the novel PoP technology. The optimization tool works as a Decision Support Tool to consult and support end-users for research and deployment (plating shops) purposes. The mathematical formulation of the DST is presented in Table 8 and offers alternative expressions for the objective function supporting the different user-defined design needs. Specifically, each objective function was developed as a linear estimator of cost, environmental, or safety indexes, as well as a combination of all three indexes expressing a holistic view of Safe and Sustainable by Design (SSbD) performance. The optimization model involves non-linear constraints, and the problem can be solved using NLP solvers, such as the Generalized Reduced Gradient that was used in this work.

The parameters of the DST in Table 8 were prepared for plating on ABS. The constraints of the optimization model include 10 equalities related to process unit operations and property prediction models, as well as two extra constraints (last row of Table 8) that embed user-defined adhesion (${AD}^{spec}$) and thickness (${TH}^{spec}$) specifications of the metal coating. The three user-defined inputs for the execution of the DST include:

1. The number of items and surface [cm^2^] per item for processing;
2. The baths’ volume [lt] considering the same volume for all processing steps (etching, activation, reduction, electroless plating and electroplating); and
3. The adhesion [MPa] and thickness [μm] specifications of the plated items.

The outcomes of the DST include the optimal conditions for each processing step as summarized in Table 9. The upper and lower bounds of process variables are considered based on the operation boundaries tested in experimental runs to avoid uncertainty due to extrapolation of conditions.

Alternative objective functions were formulated to express economic, environmental and safety impacts or combined insights of all three impacts serving a holistic SSbD approach driving the optimization of the PoP process design variables. The impacts were identified for materials and energy flows related to the consumption of (a) H_2_O_2_ and H_2_SO_4_ in etching, (b) NiA_2_ in activation, (c) NaBH_4_ in reduction, and (d) nickel and energy (electricity) in electroplating. Each individual impact is expressed as a linear approximation of the respective flow, $F_{j}$ [kg or kWh]—where $Set\;J:j=\left[H_{2}O_{2},H_{2}SO_{4},{NiA}_{2},NaBH_{4},Ni,electricity\right]$—and the unit impact factors (parameter $a_{j}$) of the flow j. The materials and energy flows, $F_{j}$, are computed by the process and property models involved in the constraints of Table 8. The unit impact factors $a_{j}$ related to economic ($a_{j}^{cost}$), environmental ($a_{j}^{envi}$), and safety ($a_{j}^{safe}$) were identified in the literature and public databases or were computationally approximated. Materials and energy unit costs [€/kg or €/kWh] can be found in commodities price databases. The normalized and weighted environmental impact scores [mPt/kg or mPt/kWh] were extracted by public and commercial life cycle inventories (e.g., ecoinvent, simapro). The normalized safety scores can be computed based on combined scores for human, environmental and physical hazards found in ECHA or predicted by QSAR models [30]. As a result, four objective functions were developed (Equation (25)) for each optimization problem to minimize cost (${Obj}^{cost}$), environmental (${Obj}^{envi}$), safety (${Obj}^{safe}$) and SSbD (${Obj}^{SSbD}$) impacts.

(25a) $$Min\;{Obj}^{cost}=\sum_{j\in J}a_{j}^{cost}\cdot F_{j}$$

(25b) $$Min\;{Obj}^{envi}=\sum_{j\in J}a_{j}^{envi}\cdot F_{j}$$

(25c) $$Min\;{Obj}^{safe}=\sum_{j\in J}a_{j}^{safe}\cdot F_{j}$$

(25d) $$Min\;{Obj}^{SSbD}=w_{cost}\cdot f\left({Obj}^{cost}\right)+w_{envi}\cdot f\left({Obj}^{envi}\right)+w_{safe}\cdot f\left({Obj}^{safe}\right)$$

where $w_{cost}$, $w_{envi}$, and $w_{safe}$ are weights used to adapt the contribution of individual objectives to the whole SSbD objective.

**Figure 14:**
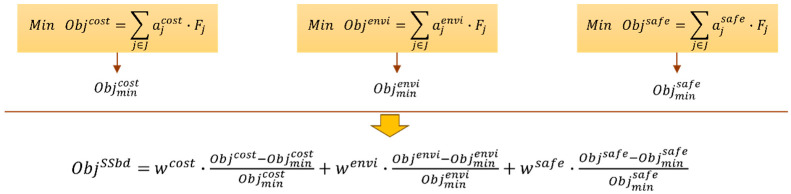
SSbD objective modeling approach.

To incorporate the individual objectives ${Obj}^{cost}$, ${Obj}^{envi}$ and ${Obj}^{safe}$ in the objective function that estimates the SSbD criterion, a normalization technique was used. It is noteworthy that solving for “$Min\;{Obj}^{SSbD}$” will not necessarily simultaneously achieve the minimum feasible goals for the other objectives (e.g., for cost, environmental and safety). Moreover, the SSbD objective needs to appropriately match the different scales of the other three objectives to ensure their comparable contribution. For this purpose, the individual objectives (cost, environmental and safety) are normalized with respect to the distance of their actual and minimum feasible values (${Obj}_{min}^{cost}$, ${Obj}_{min}^{envi}$, ${Obj}_{min}^{safe}$), which are estimated by first solving the individual economic, environmental and safety optimization problems as shown in Figure 14. Next, the SSbD objective is formulated as the sum of the weighted normalized individual objectives. In this work, equal weights and contributions of each objective were considered, namely $w_{cost}=w_{envi}=w_{safe}=1/3$.

### 3.3. Sensitivity Analysis of Key Variables

A sensitivity analysis was performed to address the impacts of the key independent variables of unit operations on the key output specifications. The selected input variables include the baths’ concentrations in etching, activation and reduction processing stages and the applied current in electroplating. The analysis was performed by separately varying each input variable and keeping other design conditions constant.

**Figure 15:**
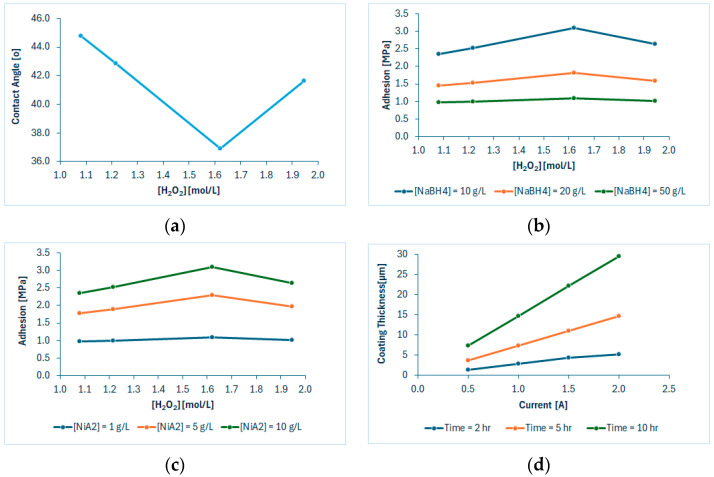
Sensitivity analysis results of (**a**) contact angle vs. piranha solution (at t = 120 s), (**b**) adhesion vs. piranha solution at 1, 5 and 10 g/L of NiA_2_, (**c**) adhesion vs. piranha solution at 10, 20 and 50 g/L of NaBH_4_, and (**d**) coating thickness vs. applied current at 2, 5 and 10 hr of electroplating.

In Figure 15a, high contact angle values were observed at a low piranha solution, indicating an inadequate formation of surface hydrophilic groups and interconnected cavities due to mild etching conditions. Similarly, high contact angles at high H_2_O_2_ concentrations indicate aggressive and uncontrolled etching that entails inadequate surface area, and, thus, low surface concentrations of hydrophilic groups. Instead, minimum contact angle values can be achieved around 1.6 mol/L of H_2_O_2_ in etching solution—equivalent to a 1:5 piranha solution (H_2_O_2_-H_2_SO_4_)—indicating a balanced formation of hydrophilic groups and surface area that is capable of serving the maximum feasible surface concentrations of potentially functionalized sites. This is evidenced by Figure 15b,c, where maximum adhesion values are also observed around a 1:5 piranha solution. Moreover, in Figure 15b, the higher the activation bath concentration, the higher the observed adhesion values due to more efficient surface chemisorption of nickel ions. Conversely, in Figure 15c, adhesion is favored by milder reduction conditions due to undesired side reactions that affect reduction and/or exhibit some kind of inhibition on nucleation sites. In Figure 15d, higher applied currents and electroplating times apparently favor electrodeposition, resulting in higher coating thicknesses. Moreover, the lines’ slopes indicate the higher impact of the electroplating time compared to that of the applied current in the formation of thicker coatings.

Overall, the sensitivity analysis highlights trends of promising operations toward high adhesion and thickness goals. These include 1:5 piranha solutions in the etching bath, 10 g/L of NiA_2_ in the activation bath, 10 g/L of NaBH_4_ in the reduction bath, and low current implementation (e.g., 0.5 A) for long electroplating processing. Nevertheless, these trends should not be interpreted as optimal operating conditions, as they are derived from a one-factor-at-a-time sensitivity analysis that does not capture the combinatorial interactions and effects among process variables and their impacts on sustainability criteria. The design variables exhibit strong combinatorial influences on the environmental, economic and safety criteria, including materials consumption, energy demand, and process efficiency. Therefore, these trends are better understood as directional insights governed by the unit operation and property models, which define the attainable region of the optimization problem. The model equations underlying these trends should be treated as constraints of the optimization problem, which is governed by the problem objectives, namely the individual or combined (SSbD) criteria.

### 3.4. Desktop Software for PoP Industry

**Figure 16:**
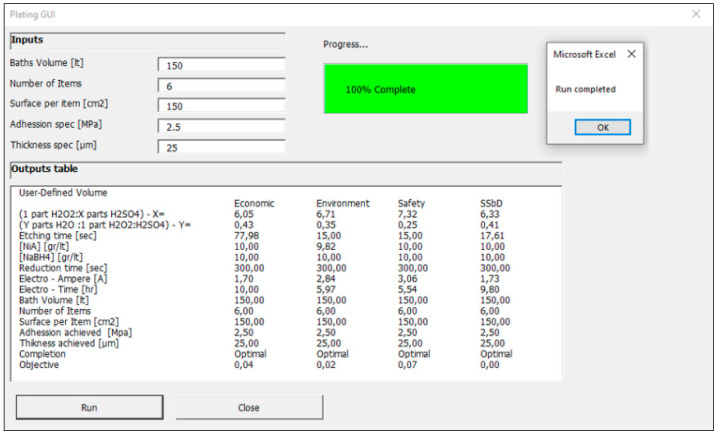
User-friendly interface of DST including user-defined input cells, monitoring of optimization results and DST completion signals.

In the course of the FreeMe project [19], a BETA version of the developed DST was constructed as a desktop software application. The program offers a GUI (Figure 16), and the optimization problem can be locally run by the user. A user-friendly interface is used to insert the problem inputs, namely the bath volume, the number and surface of items, and the adhesion and thickness specifications. The user can simply run the program, and all variables listed in Table 9 are shown on the screen of the program with respect to each objective function (economic, environmental, safety and SSbD).

### 3.5. Case Study

The beta DST version was demonstrated on a case study that reflects typical requests of the PoP industry, including bath volumes of 300 L, plating of a small set of 6 ABS plastic items of 150 cm^2^ each and specification goals of 2.5 MPa for adhesion and 25 μm for the thickness of the copper coating. Accordingly, the molecular weight and the density of copper were updated in equations estimating the mass and thickness of the coating.

Upon repeating the implementation and solving of the optimization model, a long plateau of alternative optimal solutions was identified. This phenomenon appears for all four objectives and is due to trade-offs that appear among the process variables of all the processing steps (etching, activation, reduction and plating). For example, if it is decided to consume more piranha in the etching step, thereby achieving higher surface concentrations of hydrophilic groups, then this effort can be offset by milder activation steps, achieving the same number of nucleation sites in the end. This is also justified by the results of two different runs (Run1 and Run2) in Table 10.

In Run2, the use of a different piranha concentration from Run1 returns a 10% lower contact angle than Run1, which is equivalent to a 21% higher surface concentration of hydroxyl groups ([OH]^1^) for Run2. In this scope, the model decreases by 32% the applied [NiA_2_] in Run2 compared with that of Run1 to reach the same number of functionalized sites ([Ni^2+^]) and to ensure the same adhesion specification of 2.5 MPa in both cases. The extent of such trade-offs among alternative solutions that serve the same specification outputs is driven by the applied objective function each time and explains how much each variable can be favored at the cost of another variable toward minimization of an objective. This phenomenon enables plating shops to set up the new PoP process considering a wide operating window of alternative solutions. The ability to tune the processing steps in different ways while achieving the same adhesion and specification targets at optimal performance offers valuable flexibility for practitioners, allowing them to adapt operations to the specific everyday plan, bath specifications and supplies.

To better understand tuning flexibility across a range of feasible alternative solutions, the DST was used to generate a set of 10 indicative solutions. It is noteworthy that all runs achieved optimality of the objectives, satisfying the desired constraints. This verifies the fact that the requested design is feasible within the allowed lower-upper bounds of process variables considered in the model. Moreover, this verifies the capacity of the algorithm to overcome bottlenecks, like non-linear programming initialization and solving, as well as calculation and runtime errors. Table 11 summarizes the *average values* of the independent process design variables observed among the 10 solutions, along with their operating ranges ([min value, max value]), within which the operators can adapt their design procedure. Different values within the resulting operating ranges (Table 11) should be appropriately selected to ensure satisfaction of all constraints and optimal performance. It is also normal that different average values and ranges are observed in Table 11 with respect to each criterion (economic, environmental, safety and SSbD) due to the different effects of each process variable on each objective. These effects are determined by the indicators ($a_{j}^{cost}$, $a_{j}^{envi}$, $a_{j}^{safe}$) and weights ($w_{cost}$, $w_{envi}$, $w_{safe}$) used in each objective function in Equations (25a)–(25d).

## 4. Discussion

This work provides a new and comprehensive modeling approach to model, simulate and optimize all processing steps of an emerging Cr^6+^-free and Pd-free PoP technology. The technology replaces Cr^6+^ solutions with piranha solutions for etching plastics, while Pd/Sn colloid is replaced with nickel salts and NaBH_4_ for activation and reduction purposes.

The new PoP technology was developed in the course of the FreeMe project considering demonstration components widely used in home appliances and the automotive industry. Metal-plated ABS is used in door frames, handles and the buttons of washing machines, providing enhanced corrosion resistance, improvement of external appearance and insulation against electricity and heat. Similarly, several interior and exterior car components (e.g., instrument frames, steering wheel trim, door handles, logos, and emblems) are made from plated ABS because of its light weight, wear resistance, and aesthetic appeal. Accordingly, an average, but still conservative, minimum adhesion specification of 2.5 MPa was horizontally considered for all applications, and the experimental and modeling results justified the capability of the new technology to be adopted by the involved industries.

The minimum contact angle of 28° was achieved by etching with 1:5 H_2_O_2_-H_2_SO_4_ piranha solutions and at relatively low etching times (only 2 min), while the highest adhesion observed was at 8.85 MPa (with an average of >3 MPa), when operating at selected conditions (1:5 H_2_O_2_-H_2_SO_4_ in etching, 10 g/L of NiA_2_ in activation and 10 g/L in reduction). Comparatively, etching of ABS with MnO_2_–H_2_SO_4_ colloid for 20 min reached contact angle values of 38° [8], while etching with KMnO_4_ for 20 min decreased the contact angle to 40.51°, reaching an adhesion value of 2.73 MPa [7]. Similarly, promising etching performance was observed by MnO_2_-H_3_PO_4_-H_2_SO_4_ colloid, achieving a minimum contact angle of 29.6° (at 10 min etching) and adhesion up to 1.33 kN/m, while the typical adhesion achieved by Cr^6+^ solutions is 1.42 kN/m [11].

## 5. Conclusions

The models developed in this work include:

▪ A new data-driven contact angle prediction model as a function of piranha solution and etching time, serving as an equivalent of an etching kinetic model;
▪ A new data-driven modeling approach that relates surface concentration of hydrophilic groups with contact angle;
▪ A new regression model to estimate the extent of activation as a function of applied nickel salts concentration;
▪ A new reduction kinetics model based on state-of-the-art principles;
▪ A new regression adhesion prediction model as a function of surface concentration of reduced nickel sites; and
▪ A coating thickness estimation model based on state-of-the-art principles.

A contact angle property prediction model is proposed to embed the complex reaction mechanisms of ABS polymer oxidation as a function of the oxidizer concentration and the etching time in a simplified and adaptable formulation. The modeling approach is expected to offer an improved fitting performance for different polymers that follow similar etching mechanisms (e.g., PC-ABS or similar resins). Regarding the ABS tested, a wide range of operating conditions was experimentally investigated, enabling process optimization within that range. Operating conditions outside the tested piranha concentration range (1:4–1:10 H_2_O_2_:H_2_SO_4_) are expected to perform either uncontrolled or weak etching, resulting in poor surface properties. When the model needs to be extrapolated to other conditions and polymers, then three simple experimental data—piranha concentration, etching time and contact angle—can be fitted for model calibration. The correlation between contact angle and surface concentration of hydroxyl groups considers surface morphologies with micro-/nano-cavities and contact angles driven by the hydrophilicity of such groups, while minor contributions are expected by other chemical groups. The nickel chemisorption model for the activation stage offers an improved fitting performance following a Langmuir-like formulation. The reduction kinetics model was iteratively developed by fitting it across the range of tested concentrations, allowing it to capture the different responses of the reaction system; for example, conditions under which the desired or competing side reactions are favored at low or high NaBH_4_ concentrations. The adhesion model embeds strengths originating from chemical bonding between nickel elements used as anchoring points and for the first electroless layer (Ni-P), as well as additional contributions of mechanical interlocking due to surface morphology. The model offers predictions within weak (<1 MPa) and stronger (up to 8 MPa) adhesion values; however, high reliability predictions are centered around modest predictions, e.g., in the range of 2–4 MPa. The thickness modeling concept follows typical electroplating principles, including theoretical mass and time- and current-dependent growth rates that can be easily adapted to nickel, copper, or other metals used. All models were developed and tested based on experiments that serve a wide range of operating conditions for each processing stage, where optimal conditions are expected and identified. This is justified by the optimal solutions proposed by the DST, which lie within the upper and lower boundaries (Table 11).

All models were constructed as a DST for local use (on a desktop) through a user-friendly interface to assist plating shops and researchers in experimentation, analysis, and optimization of technology variables. The model also supports different economic, environmental, safety and SSbD objectives and revealed important insights about setting up the PoP process across a flexible window of tunings, ensuring user-defined specifications for adhesion and thickness. The solving procedure per optimization problem could be solved in less than 1 s, or a few seconds for more demanding cases, e.g., for very high adhesion specifications. In this scope, the DST could be incorporated and interconnected with ICT and control systems for real-time monitoring, optimization and decision-making, supporting and enhancing Industry 4.0 applications toward SSbD manufacturing.

## Figures and Tables

**Table 1:** Chemical composition and operating conditions of electroless Ni-P plating bath.

Compound	Concentration (g/L)
NiSO_4_·6H_2_O (≥98%)	32
Na_3_C_6_H_5_O_7_·2H_2_O (≥99%) (sodium citrate dihydrate)	20
NaPO_2_H_2_ (≥98%)	28
NH_4_Cl (≥99.5%)	25
NH_4_OH (25% aqueous solution)	Until pH = 9
**Operating Conditions**	
Temperature	45 °C
pH	9 (adjusted with NH_4_OH)

**Table 2:** Chemical composition and operating conditions of electroplating nickel Watts bath.

Compound	Concentration (g/L)
NiSO_4_·6H_2_O (≥98%)	300
NiCl_2_·6H_2_O (≥99%)	35
H_3_BO_3_ (≥95%)	40
Saccharin (C_7_H_5_NO_3_S) (≥%)	2
Sodium dodecyl sulphate, SDS (NaC_12_H_25_SO_4_)	2.5
NH_4_OH (25% aqueous solution)	Until pH = 4.4
**Operating Conditions**	
Temperature	50–60 °C
pH	4.4 (adjusted with NH_4_OH)

**Table 3:** Experimental results of etching ABS substrates at different conditions (piranha solutions and times) and achieved contact angle after etching.

Piranha H_2_O_2_-H_2_SO_4_ [*v*/*v*]	Etching Time [s]	Average Contact Angle [°]	Standard Deviation Contact Angle [°]
1:5	15	47.6	2.8
1:5	30	40.9	2.7
1:5	60	37.5	3.9
1:5	120	34.9	2.1
1:5	180	28.1	2.2
1:7	30	48.2	6.5
1:7	60	41.4	2.2
1:7	120	40.3	2.2
1:10	30	38.7	2.4
1:10	120	35.9	1.5

**Table 4:** Experimentally measured contact angle and calculated concentrations of hydrophilic groups before and after activation. *The standard deviation (std) of the experimental data is reported in italics*.

	Before Activation		After Activation	
[NiA_2_] [g/L]	CA^1^ [°]	[OH]^1^ (calc) [mol/cm^2^]	CA^2^ [°]	[OH]^2^ (calc) [mol/cm^2^]
1	38.7 *(std = 2.4)*	2.62 × 10^−6^	48 *(std = 5.3)*	1.69 × 10^−6^
5			52 *(std = 4.2)*	1.40 × 10^−6^
10			54 *(std = 5.2)*	1.28 × 10^−6^

**Table 5:** Surface elemental composition before and after activation.

	Before Activation		After Activation	
Element	Atomic %	Weight %	Atomic %	Weight %
Carbon	80.6	69.4	73.2	59.2
Nickel	2.7	11.4	3.5	13.9
Oxygen	16.7	19.2	19.6	21.2
Sodium			3.7	5.7

**Table 6:** Experimental results for different conditions of surface reduction stage.

[NaBH_4_] [g/L]	Reduction Time [min]	Average of Adhesion [MPa]	Standard Deviation of Adhesion [MPa]
10	1	2.44	0.54
10	5	8.85	0.88
10	10	6.89	4.29
20	1	1.71	0.46
20	5	1.36	0.17
20	10	2.48	1.19
50	1	1.33	0.29
50	5	1.2	0.14
50	10	1.6	0.36

**Table 7:** Electroplating experimental results over different current densities and time. *The standard deviation (std) of the experimental data is reported in italics*.

Surface [dm^2^]	Current Density [A/dm^2^]	Average Real Mass [g]	Time [h]	Theor. Mass [g]	Growth Rate [1/s]
0.3	1.3	0.086 *(std = 0.016)*	0.5	0.214	2.88 × 10^−4^
0.3	1.4	0.107 *(std = 0.005)*	2	0.920	1.71 × 10^−5^
0.3	1.5	0.326 *(std = 0.023)*	2	0.985	5.58 × 10^−5^
0.3	1.7	0.166 *(std = 0.018)*	0.5	0.279	5.03 × 10^−4^

**Table 8:** DST optimization model.

Min obj = f(economic and/or environment and/or safety index)	
s.t.	
▪ $CA={CA}^{0}\cdot exp\left(\frac{-B\cdot t_{etching}}{t+1}\right)$ ▪ $B=exp\left(A^{\prime }+\frac{B^{\prime }}{\left[H_{2}O_{2}\right]}+C^{\prime }\cdot ln\left(\left[H_{2}O_{2}\right]\right)+D^{\prime }\cdot {\left(\left[H_{2}O_{2}\right]\right)}^{E^{\prime }}\right)$ ▪ $ln\left(480.45\cdot {\left[OH\right]}^{1}+1\right)=-0.0818\cdot \left(CA-{CA}^{0}\right)$	Etching stage—Contact angle and hydrophilic groups surface concentration
▪ ${\left[OH\right]}^{2}=\frac{{\left[OH\right]}^{1}}{0.1057\cdot \left[{NiA}_{2}\right]+1}$ ▪ $\left[{Ni}^{2+}\right]=\frac{{\left[OH\right]}^{1}-{\left[OH\right]}^{2}}{2}$	Activation stage—Surface concentration of formed Ni^2+^ sites
▪ ${\left[{Ni}^{2+}\right]}^{t+1}={\left[{Ni}^{2+}\right]}^{t}-\frac{k2\cdot \left[{Ni}^{2+}\right]\cdot \left[NaBH_{4}\right]}{1+k3\cdot {\left[NaBH_{4}\right]}^{2}}\cdot \Delta t_{reduction}$	Reduction stage—Surface concentration of Ni^0^ nucleation sites
▪ ${AD}^{corrected}=\frac{{AD}^{max}\cdot L\cdot e^{\frac{AD-1.0212}{2.279}}}{1+L\cdot e^{\frac{AD-1.0212}{2.279}}},where\;AD=\frac{{AD}^{max}\cdot N\cdot \left[{Ni}^{0}\right]}{1+N\cdot \left[{Ni}^{0}\right]}$ ▪ ${mass}^{real}=\frac{I\cdot S\cdot t\cdot 58.69}{2\cdot 96485\;}\cdot \left(1-e^{-gr\cdot t_{electro}}\right)$ ▪ $gr=0.0093\cdot I^{2}-0.0277\cdot I+0.0207$ ▪ $TH=\frac{{mass}^{real}}{\rho \cdot S}$	Plating stage—Achieved adhesion and thickness specifications
▪ $AD\geq {AD}^{spec}$ ▪ $TH={TH}^{spec}$	User-defined specifications—Additional constraints

**Table 9:** Optimization variables and generated results of DST.

Variable Name	Optimization Variable	Lower-Upper Bounds of Model Variables
Etching process		
$\left[H_{2}O_{2}\right]$	1:$X_{1}$ ratio of H_2_O_2_:H_2_SO_4_	$X_{1}$ = [4, 10]
Piranha bath dilution	$X_{2}$ parts of H_2_O per part of piranha	$X_{2}$ = [0, U] *
$t_{etching}$	$X_{3}$ s	$X_{3}$ = [15, 120]
Activation process		
*[* ${NiA}_{2}$ *]*	$X_{4}$ g/L of NiA_2_	$X_{4}$ = [1, 10]
Reduction process		
*[NaBH_4_]*	$X_{5}$ g/L of NaBH_4_	$X_{5}$ = [1, 50]
$t_{reduction}$	$X_{6}$ s	$X_{6}$ = [1, 300]
Electroless plating		
Fixed recipe and conditions		
Electroplating		
*Current (* I *)*	$X_{7}$ A/cm^2^ **	$X_{7}$ ≥ 0
$t_{electro}$	$X_{8}$ h	$X_{8}$ ≥ 0
PoP specifications and goals		
Adhesion achieved	$X_{9}$ MPa	$X_{9}$ ≥ adhesion spec
Thickness achieved	$X_{10}$ μm	$X_{10}$ = thickness spec
Objective	Index	

* A large number; ** 18 volts are applied in bath.

**Table 10:** Comparative analysis of process optimization results.

Process Variable	Run1	Run2	$\mathrm{Trade}-\mathrm{Offs}\;\left(\frac{Run2}{Run1}-100\%\right)$
H_2_O_2_:H_2_SO_4_ (Piranha) [*v*/*v*]	1:8.3	1:5.4	
Piranha:Water [*v*/*v*]	1:0.1	1:0.7	
Etching time [s]	15	15	
Contact angle (°)	43.1	38.6	−10%
[OH]^1^ [mol/cm^2^]	4.59 × 10^−6^	5.58 × 10^−6^	+21%
[NiA_2_] [g/L]	10.0	6.8	−32%
[Ni^2+^] [mol/cm^2^]	1.26 × 10^−6^	1.26 × 10^−6^	
[NaBH_4_] [g/L]	10.0	10.0	
Reduction time [s]	300	300	
[Ni^0^] [mol/cm^2^]	1.8 × 10^−10^	1.8 × 10^−10^	
Adhesion [MPa]	2.50	2.50	0%
Objective (economic)	4 × 10^−2^	4 × 10^−2^	0%

**Table 11:** Average values (in italics) and operating ranges (in brackets) of selected solutions for a set of 10 runs.

Objective	Economic	Environmental	Safety	SSbD
H_2_O_2_:H_2_SO_4_ (Piranha) [*v*/*v*]	*1:6.1* [1:4.0, 1:9.4]	*1:7.3* [1:4.0, 1:9.8]	*1:6.7* [1:4.3, 1:9.1]	*1:6.8* [1:4.0, 1:9.0]
Piranha:Water [*v*/*v*]	*1:0.3* [1:0.0, 1:0.8]	*1:0.2* [1:0.0, 1:0.5]	*1:0.2* [1:0.0, 1:0.6]	*1:0.1* [1:0.0, 1:0.3]
Etching time [s]	*65* [15, 116]	*84* [18, 120]	*40* [15, 98]	*37* [15, 94]
[NiA_2_] [g/L]	*7.5* [5.9, 10.0]	*8.1* [6.0, 10.0]	*9.2* [6.2, 10.0]	*9.6* [6.0, 10.0]
[NaBH_4_] [g/L]	*10.0* [10.0, 10.0]	*10.4* [10.0, 12.3]	*10.0* [10.0, 10.0]	*10.0* [10.0, 10.0]
Reduction time [s]	*300* [300, 300]	*300* [300, 300]	*300* [300, 300]	*300* [300, 300]
Current [A]	*2.21* [1.70, 3.06]	*2.11* [1.70, 3.06]	*2.50* [1.70, 3.06]	*1.98* [1.70, 3.06]
Electroplating time [h]	*8.03* [5.54, 10.00]	*8.47* [5.54, 10.00]	*7.28* [5.54, 10.00]	*9.02* [5.54, 10.00]
Adhesion [MPa]	*2.5*	*2.5*	*2.5*	*2.5*
Thickness [μm]	*25.0*	*25.0*	*25.0*	*25.0*

## Data Availability

The raw data supporting the conclusions of this article will be made available by the authors on request.

## Abbreviations

The following abbreviations are used in this manuscript:

PoP	Plating on Plastics
SSbD	Safe and Sustainable by Design
ABS	acrylonitrile–butadiene–styrene
DST	Decision Support Tool
NiA_2_	nickel acetate
CA	contact angle
CA^0^; CA^1^; CA^min^	contact angle of untreated polymer; at state 1; and minimum observed
AD; AD^max^	adhesion; and maximum observed
TH	thickness
gr	growth rate of the electroplating process
t	time
calc	calculated
I	current
S	surface
MW	molecular weight
n	number of electrons
F	Faraday constant
J	set of considered flows affecting the objective function (including elements j)
Obj^cost^; Obj^envi^; Obj^safe^	objective function estimating cost; environmental; and safety indexes
